# A comprehensive narrative review of challenges and facilitators in the implementation of various HPV vaccination program worldwide

**DOI:** 10.1002/cam4.6862

**Published:** 2024-01-11

**Authors:** Sumit Aggarwal, Pragati Agarwal, Nivedita Gupta

**Affiliations:** ^1^ Division of ECD, Indian Council of Medical Research New Delhi India

**Keywords:** Cervarix, cervical cancer, Gardasil, human papilloma virus, vaccination

## Abstract

**Introduction:**

Cervical cancer has been considered as one of the most common cancers in women (15–44 years) globally, but the advent of the human papilloma virus (HPV) vaccine has raised the anticipation that eradication of cervical carcinoma might be achieved in the near future as several prophylactic cervical carcinoma vaccines have already been currently licensed in various countries. Countries should devise strategies, practices and policies to attain and sustain higher levels of HPV immunization coverage as still 68% countries have introduced HPV vaccine in their national immunization programs even after 17 years following the licensure of the first prophylactic HPV vaccine.

**Methodology:**

A comprehensive literature analysis was conducted using various databases and search engines, to include the most relevant research articles and data available and critically discussed the operational gaps that need to be answered to achieve adequate coverage of HPV vaccination.

**Results:**

The present review highlights the existing HPV vaccination strategies, unmet needs and challenges needed to be addressed for proper implementation framework as well as the collaborations required to achieve decent vaccination coverage. Well‐coordinated vaccination strategy with focus on adolescent girls and if possible, boys can lead to dramatic impact on disease reduction around the world.

## Graphical abstract

Practices and policies related to human papilloma virus (HPV) vaccination need to be improved to achieve better vaccination coverage. The present review highlights the existing HPV vaccination strategies, unmet needs and challenges needed to be addressed for proper implementation framework as well as the collaborations required to achieve decent vaccination coverage.

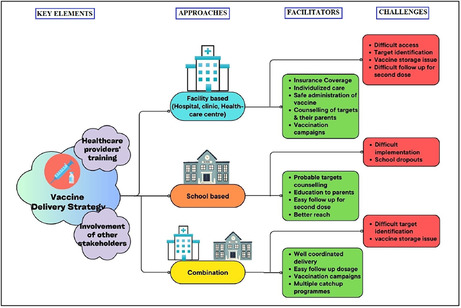

## 1 INTRODUCTION

Cervical carcinoma is an important public health issue and the second most abundant cancer in women aged 15–44 years in India and the fourth most common globally, with more than 75% of its cases being diagnosed at advanced clinical stage, that is, with low prospects of survival. According to the World Health Organization (WHO), more than 0.6 million confirmed new cases of cervical carcinoma were observed worldwide in 2020, and >0.34 million deaths were ascribed to its malignancy.^1^ Approximately 90% of the cases and deaths occurred in low‐ and middle‐income countries (LMICs) globally in 2020,^2^ primarily due to delayed diagnostic services, constrained access to preventative procedures, and treatment services (e.g., surgery, radiotherapy, and chemotherapy). The situation is even frightening in rural areas, where common women lack awareness and knowledge regarding the threats of cervical cancer, and health care means are also scarce.^3^ The collective risk of developing cervical carcinoma starts rising from the age of 30 to 34 years and peaks at around 55 years.^4^ While cervical carcinoma prevalence is nowadays declining globally, it still poses a significant burden.

The most critical risk factor for the development of intraepithelial neoplasia and invasive cervical carcinoma is persistent infection by a subclass of human papillomavirus (HPV).^5^ HPV16 and 18 are allied to more than 50% of high‐grade cervical carcinomas. HPV16 is predominantly correlated to squamous cell carcinomas (SCC), whereas HPV18 is mostly diagnosed in adenocarcinomas.^6^ Furthermore, population projections propose that the declining incidence of cervical carcinoma may not be enough to duck substantial increase in the number of females affected during the coming decades.^7^ HPV is primarily transmitted via sexual interaction, and most people gets infected soon following the commencement of sexual activity, while some may be repeatedly infected. Although most HPV infections and precancerous lesions clear up spontaneously, there always remains a risk that the infection may develop into chronic and precancerous lesions may advance into invasive cervical cancer.^8^ Cervical carcinoma develops in 15–20 years following infection in normal women, while 5–10 years in females with weakened immune systems, for instance women with untreated HIV infection.

Although several methods for preventing cervical cancer exist, prevention through vaccination has emerged as the most effective option.^4^ The advent of HPV vaccines has raised the prospect that the eradication of cervical carcinoma might be conceivable in the coming decade. Several effective and safe vaccines have been licensed globally, such as the quadrivalent GardasilTM, nonavalent Gardasil9, both by Merck, and the bivalent vaccine CervarixTM of GlaxoSmithKline make.^9^ However, the fragile health systems of LMICs are ineffectively equipped to face the challenging question of delivering dual doses of the vaccine to adolescent females.^10^ The WHO has called on all countries to scale up the execution of demonstrated, verified strategies and policies at priority to eradicate cervical carcinoma by 2030.^11^ However, it is evident that the cervical carcinoma vaccine is not an instant solution as it cannot substitute the essence of cervical carcinoma screening.^12^ The burden of cervical carcinoma can be significantly reduced by a careful combination of high‐coverage, affordable HPV vaccination of girls (9–14 years old), and organized and accessible screening programs.^7^ This article conducts an in‐depth review to map and characterize the types of existing evidence associated with the challenges, attitudes, as well as practices toward HPV vaccination strategies and to identify operational research gaps associated with it.

## 2 METHODOLOGY

### 2.1 Study design

To map the challenges and strategies related to HPV vaccination and identify operational research gaps, a comprehensive review was conducted. This review provides insights into the gaps and research priorities necessary for the efficient implementation of vaccination policies.

### 2.2 Search strategy and data sources

Given the current situation of cervical carcinoma and gaps in vaccination coverage, a thorough investigation of health policies for efficient vaccination strategy implementation was deemed crucial. Therefore, a comprehensive literature analysis was conducted using various databases and search engines, including Medlineplus (NLM), PubMed, and Embase databases, among others. The search incorporated keywords such as “HPV” AND “vaccination,” “HPV vaccination” AND “delivery strategies,” “HPV vaccination” AND “challenges,” “HPV vaccination” AND “coverage,” and so on. The backward and forward snowballing approach was also employed to identify additional relevant research articles. The review extensively studied published research articles, case reports, national health authority guidelines and advisories, surveillance reports, and press releases from international health organizations such as the WHO and CDC, focusing on HPV vaccination since 2015.

### 2.3 Study selection criteria

To identify pertinent literature and ensure a comprehensive review, several selection criteria were applied to the searched literature.

Inclusion criteria:

- Articles describing cervical cancer preventive and HPV vaccination strategies.
- Published grey literature.
- Articles published in English.
- Articles published from 2015 to 2023.

Exclusion criteria:

- Studies stating the facts that are not of interest or provide irrelevant information.
- Articles that were not peer‐reviewed.
- Full text not available.

A multilevel screening approach was implemented, and articles and documents that were out of context were excluded. The remaining articles underwent a thorough review by three independent investigators specializing in the fields of public health, biotechnology, and virology. This approach ensured a rigorous and in‐depth qualitative analysis of the content through an interdisciplinary perspective.

This review was conducted using the most relevant as well as comprehensive available sources and critically discussed the operational gaps that need to be answered to achieve adequate coverage of HPV vaccination.

## 3 RESULTS

### 3.1 Literature search

**FIGURE 1:**
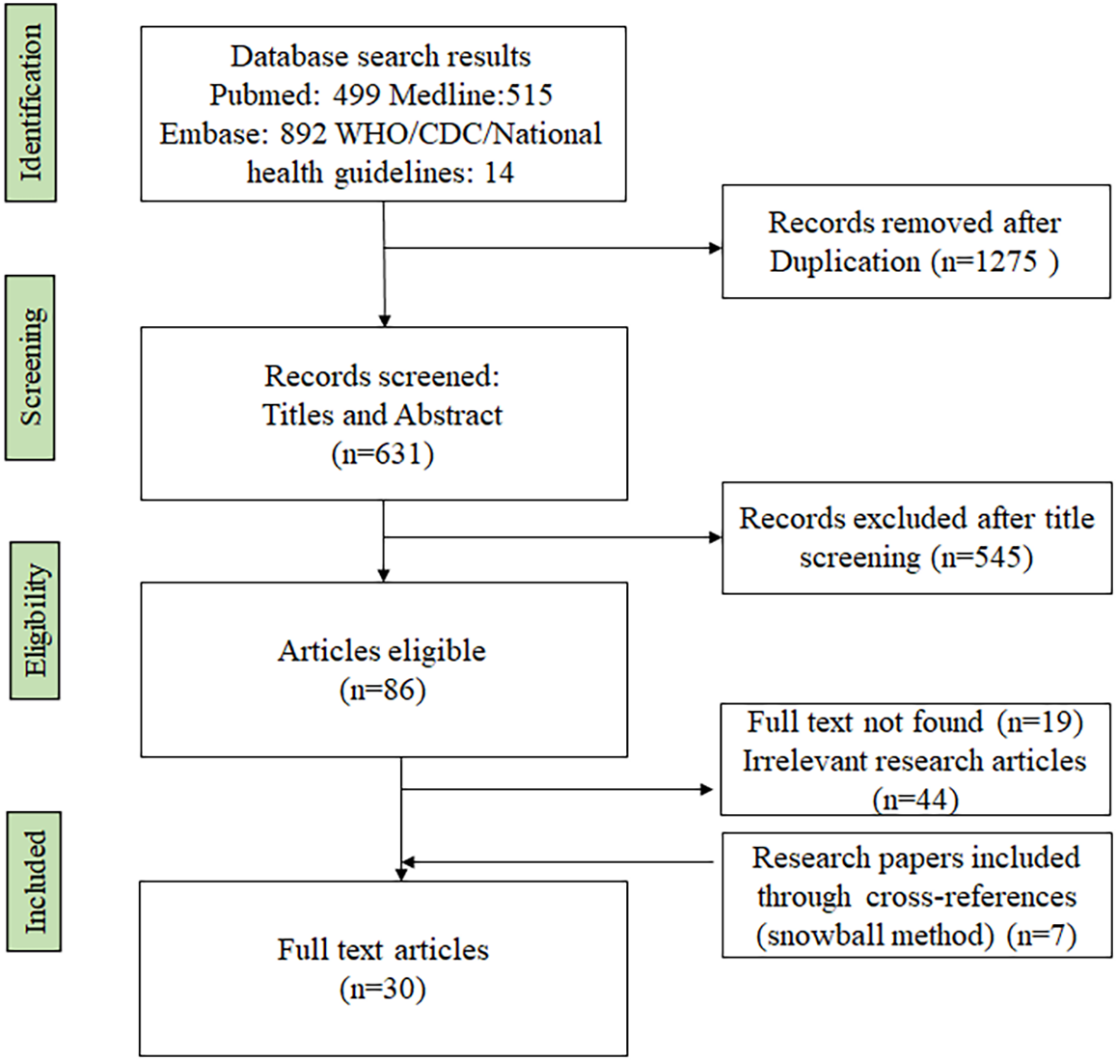
Search strategies for studies included in the review.

Primarily PubMed, Medlineplus, and Embase databases were explored for searching relevant literature with time filter to extract the articles published after the introduction of HPV vaccination drive in various nations. Overall, 1906 articles were extracted via initial searching and after eliminating duplicate articles, 631 articles met the eligibility criteria and after title and abstract screening, finally, 30 articles were selected for inclusion in the present review. Figure 1 illustrates the search strategy diagram of the identified studies.

### 3.2 Overview of included studies

A total of 30 articles were included in the present narrative review which were published in journals related to the medical and health sciences subject area. The included articles were published by researchers from 11 different countries. Among these, three studies were conducted in Switzerland, spanning from 2018 to 2023, and they focused on various aspects of HPV vaccination among the Swiss population. Additionally, two studies each were conducted in France (2023), the United States (2020, 2022), and India (2020, 2022). The majority of the articles investigated HPV vaccination knowledge, delivery strategies, determinants, and challenges as the main outcomes.

### 3.3 Available vaccines globally

Till now, four HPV vaccines have been licensed by the US Food and Drug Administration (FDA) and several other countries: a quadrivalent vaccine Gardasil TM, nonavalent Gardasil9,^13^ a bivalent vaccine CervarixTM,^9^, ^14^ and a recently developed vaccine Cecolin^15^ as listed in Table 1. All these vaccines were manufactured from the L1 proteins of HPV strains and provided protection against vulvar, vaginal, precancerous cervical lesions CIN I‐III, while the nonavalent vaccine provided protection against oropharyngeal cancer also. Other than this, several L2‐based HPV vaccine candidates such as RG1‐ VLP and CUT‐PANHPVAX have generated promising preclinical data regarding broad‐spectrum and durable prophylactic efficacy, immunogenicity in reduced dose‐regimens, and thermostability, therefore potentially simplifying and reducing manufacturing costs.^16^, ^17^

**TABLE 1:** Characteristics of available HPV vaccines.

S. No.	Feature	Gardasil	Gardasil 9	Cervarix	Cecolin
1.	Type	Quadrivalent	Nonavalent	Bivalent	Bivalent
2.	Target age group	9–26 years	9–45 years	9–25 years	9–45 years
3.	Protection against	HPV 6, 11, 16, 18	HPV 6, 11, 16, 18, 31, 33, 45, 52, 58	HPV 16, 18	HPV 16, 18
4.	Prevention of	Cervical, vulvar, vaginal, anal cancer, genital warts (condyloma acuminata), precancerous, or dysplastic lesions CIN I‐III	Cervical, vulvar, vaginal, oropharyngeal, anal cancer, precancerous, or dysplastic lesions CIN I‐III, genital warts	Cervical, vulvar, vaginal, and precancerous lesions CIN I‐III	Cervical, vulvar, vaginal, and precancerous lesions CIN I‐III
5.	Components	L1 protein from HPV 6, 11, 16, 18	L1 protein of HPV 6, 11, 16, 18, 31, 33, 45, 52, 58	L1 protein from HPV 16, 18	L1 protein from HPV 16, 18
6.	L1 Protein proportion	20/40/40/20 μg	30/40/60/40/20/20/20/20/20 μg	20/20 μg	40/20 μg
7.	Adjuvant	225 μg aluminum hydroxyphosphate sulfate	500 μg amorphous aluminum hydroxyphosphate sulfate	500 μg aluminum hydroxide, 50 μg 3‐O‐deacylated‐4′‐ monophosphoryl lipid A	208 μg aluminum hydroxyphosphate sulfate
8.	Mode of administration	Intramuscular	Intramuscular	Intramuscular	Intramuscular
9.	Dosage	2–3 dose (0, 2, and 6 months)	2‐dose (0, 6–12 months)	2‐dose (0, 5–13 months)	3 doses (0, 1, and 6 months)

Apart from these vaccines several other vaccines have been developed by countries. HPV vaccine production companies based in China specifically Walvax,^18^ China National Biotech Group is among the leading companies who is manufacturing new HPV vaccines in Asia and are in currently in advanced clinical phase. As a promising progress in its fight against cervical carcinoma, India has recently launched its first locally manufactured option of HPV vaccine that was produced combinedly by the Serum Institute of India, Pune and Department of Biotechnology, Govt. of Biotechnology and approved by Drugs Controller General of India. Additionally RGVax, a chimeric HPV VLP (PathoVax LLC); TG4001 (Transgene), VGX‐3100, and INO‐3107 (Inovio) are other potential vaccine candidates which are in the clinical phase.

### 3.4 Public health approaches adopted for HPV vaccination in various countries

Global strategies for cervical cancer elimination aim to accomplish complete vaccination of 90% of adolescent females by the age of 15 years. WHO recommends the inclusion of HPV vaccines in national immunization programs (NIPs) considering the prevention of cervical carcinoma and other HPV‐associated diseases as a public health priority. France, Monaco, Switzerland, and the United States were among the first countries to include HPV vaccines in their NIPs back in 2006.^19^ Various strategies have been employed by countries for HPV vaccination as per need, and resources. Primarily three major strategies are vaccine delivery at health care facilities, outreach initiatives, for example, at school, and a combination of these two. While most of the high‐income countries (HICs) are adopting either school‐based or facility‐based approaches majorly, while LMICs and low‐income countries (LICs) have predominantly adopted combination of these two or school‐based delivery strategies. Success of these initiatives largely depends on health communication strategies, as effective health communication plays a crucial role in the successful implementation of HPV vaccination and can also be utilized to deliver various other health messages. Subsequently, here we have summarized the status, implementation strategies, challenges, and cope up mechanism for HPV vaccination in various countries.

#### 3.4.1 Facility‐based delivery

Facility‐based delivery of vaccination is the practice of providing vaccines to the target individuals in a health care facility, such as a hospital, clinic, or health center. United States was among the first few countries to include HPV vaccine in NIP in 2006, who had adopted facility‐based vaccine delivery strategy (2‐dose regimen) and was able to achieve the current vaccination rate (i.e., 73%: first dose; 50%: second dose; 2022). Immunization was provided through a variety of settings, including health centers, health care provider's office and doctor's clinics. 2017 onward, only nonavalent Gardasil9 is available in the country. To improve vaccine uptake among young adults US government organized leveraging community vaccination campaigns, elimination of cost barriers, by covering it in insurance and improved education of adult primary care clinicians regarding the risk of HPV‐related carcinomas.^20^

France also introduced HPV vaccine in its NIP in 2006 through facility‐based delivery strategy, but vaccination rates (48%: first dose and 42%: final dose; 2022) still continue to be suboptimal there and the coverage is among the lowest in Europe as they made it mandatory to be prescribed by a general practitioner. Their vaccination schedule requires 2–3 doses (based on the type of vaccine) and in 2019 vaccine recommendations were extended to boys also. From January 01, 2021, the government has provided 65% reimbursement of the vaccine cost through French Health Insurance.^21^, ^22^ Information issue about the vaccine has been acknowledged as the main barrier to the vaccination, while the other major hurdle was the concern about vaccine safety. Low coverage in facility‐based vaccination is assumed to be due to challenges in vaccine administration and barriers to HPV vaccination at health centers, also as recorded through questionnaire “Not vaccinating at school” was the most common reason for missing shots.^23^

#### 3.4.2 School‐based delivery

School‐based vaccination is the delivery of vaccines to school‐aged children using the school as a venue for delivery via a variety of providers, including nurses, doctors, or community health workers.^24^ In developed countries, for instance Australia, Canada, and Sweden, who opted for the school‐based HPV vaccine delivery strategy, vaccination coverage remained adequate and sustained.^25^ Although global experience exemplifies that school‐based vaccination programs are competent, but may be compromised by an array of aspects including parents' knowledge and attitude, teachers' and health workers' misinformation and disengagement and negative media coverage.^26^ A good instance of school‐based HPV vaccine delivery approach is as acquired by Brazil, where immunization initiated only in 2014, by the Unified Public Health System‐ Sistema Único de Saúde (SUS), initially, in line with HICs, vaccination in Brazil was carried out within schools with a coverage rate ~99%, later it was carried out exclusively at Primary Healthcare Centre, that is, Basic Health Units (Unidade Básica de Saúde; UBS) and thus there was a substantial decline in the coverage rate, that is, up to 77% by 2018.^27^

Japan introduced HPV vaccination in NIP in 2009 through school‐based delivery and it was covered under government funding through municipalities, but due to repeated media reports of adverse symptoms after HPV vaccination, the program was not well received, attributed to lack of knowledge and confidence about the HPV vaccine and thus the current vaccination rate is less than 8% in the country.^28^, ^29^ In contrast in Uzbekistan which is a LMIC, the present vaccine coverage rate is 99% (first dose and final dose) via school‐based delivery. With the help of WHO and UNICEF, the country developed a communication plan, which was one of the keys to the successful vaccine rollout. Formative research was carried out in advance, but it was through ongoing monitoring of the local situation that problems were spotted and measures were taken to keep the rollout on track. A parent‐teacher meeting was organized under the NIP, in which health care workers were invited to address questions related to misinformation circulating about the vaccine on social media. These interventions were possible due to monitoring, good planning and having a crisis communication plan, according to the WHO Uzbekistan team.^30^ Bringing all the stakeholders together to speak with one voice was another key factor for the success of the HPV vaccination program in Uzbekistan.^31^

#### 3.4.3 Combination/mixed delivery

A mixed delivery strategy of vaccination is a method of administering vaccines combining two or more different delivery methods to make it more convenient for recipients, or to reach a wider population. Switzerland who was among the first countries to include HPV vaccine in its NIP, implemented a canton‐wide vaccination drive via three different and complementary service approaches: in public schools, by private physicians and by the University Hospital with a coverage rate of 74% (first dose) and 71% (final dose) presently.^32^ A 2‐dose vaccination schedule (0 and 6 months) was initiated in 2012, replacing the earlier 3‐dose schedule and was extended to male individuals since 2015. Easy and free access to vaccination; well‐coordinated delivery services, inclusion at school health centers, public hospitals, and private physicians; as well as increased knowledge imparted key role in the program's success. Media interest, public promotion of the immunization program, direct mailing the vaccine vouchers, and availability to catch‐up HPV vaccination at primary care have also played key roles in the program's success.^33^, ^34^ In Spain, a fully government‐funded systematic HPV immunization in girls (aged 12–14 years) was introduced in 2007 with a projected coverage for the first dose 90% and 81% for the complete schedule in 2022. HPV vaccines are offered to all girls and boys in schools in Spain. People who missed out on the school‐based vaccination program can get vaccinated through catch‐up programs provided at primary care centers. The launch of various informative as well as awareness‐raising activities on the importance of HPV and its prevention promoted by several scientific societies and also marking March 4 as the annual International HPV Awareness Day, 2018 onward are the identified causes for successful vaccination in the country.^35^

### 3.5 Analysis of coverage of HPV vaccination achieved through various strategies globally

**FIGURE 2:**
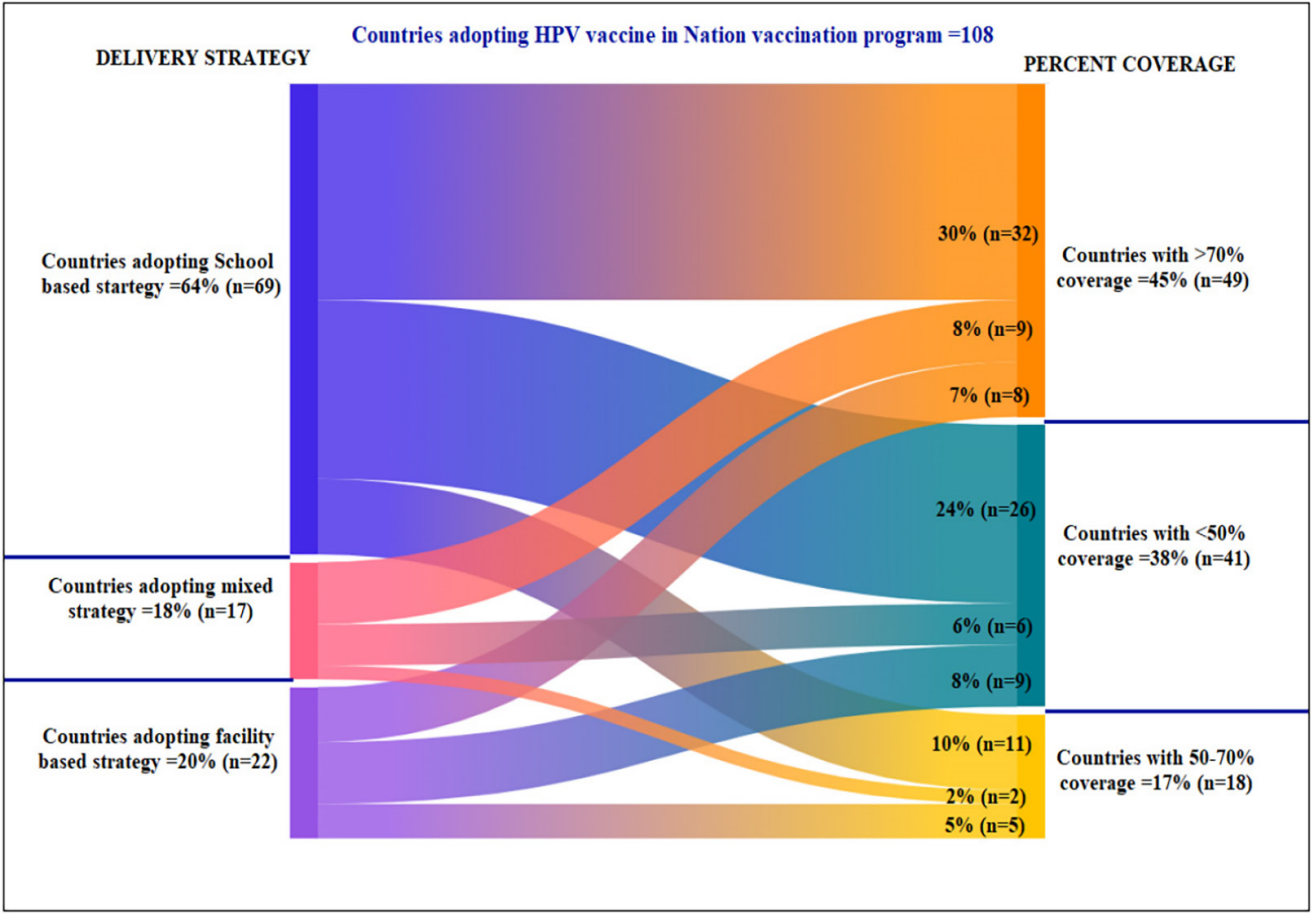
Human papilloma virus vaccination coverage in countries through various vaccine delivery strategies, as per WHO (Data are available for 108 countries implementing the program).

As per WHO,^36^ 133 countries have included HPV vaccines into their NIPs, out of which the most adopted strategy was school‐based delivery in 69 countries (64%) and out of these 32 countries (30%) were able to achieve the vaccination coverage >70% attributed to efficient communication plan, while was below 50% in almost 24% countries, who adopted the strategy (Figure 2).

Facility‐based delivery strategy was adopted by ~20%, through which 36% countries adopting the approach could achieve the coverage >70%, while vaccination coverage was below 50% in other 41% countries adopting the same approach due to implementation failure.

Mixed delivery strategy is the most successful strategy, although implemented in only 16% countries, with a coverage rate of >70% in 53% countries adopting the approach due to easy access to vaccination.

Nations should adopt strategies that are compatible with their delivery infrastructure and cold chain capacity, cost‐effective, sustainable, and capable to achieve the highest possible immunization coverage. As evident from the data the most frequently adopted approach worldwide is school‐based vaccination (~64%) with 32 out of 69 (~46%) countries adopting this strategy achieving the vaccination coverage >70%. It may be required to employ a combination of approaches to ensure access for all eligible girls. Involvement of the policy framing committees, regulatory bodies, political and financial agencies must be ensured. HPV vaccines should be introduced as part of a coordinated and comprehensive strategy in NIP in a systematic and transparent manner. Educating parents play an important role in youth’ decision‐making process and they should be equally educated regarding the benefits and importance of the HPV vaccine as well as the training of health workers about screening and vaccination should be organized.

### 3.6 Challenges faced in various vaccination strategies

**FIGURE 3:**
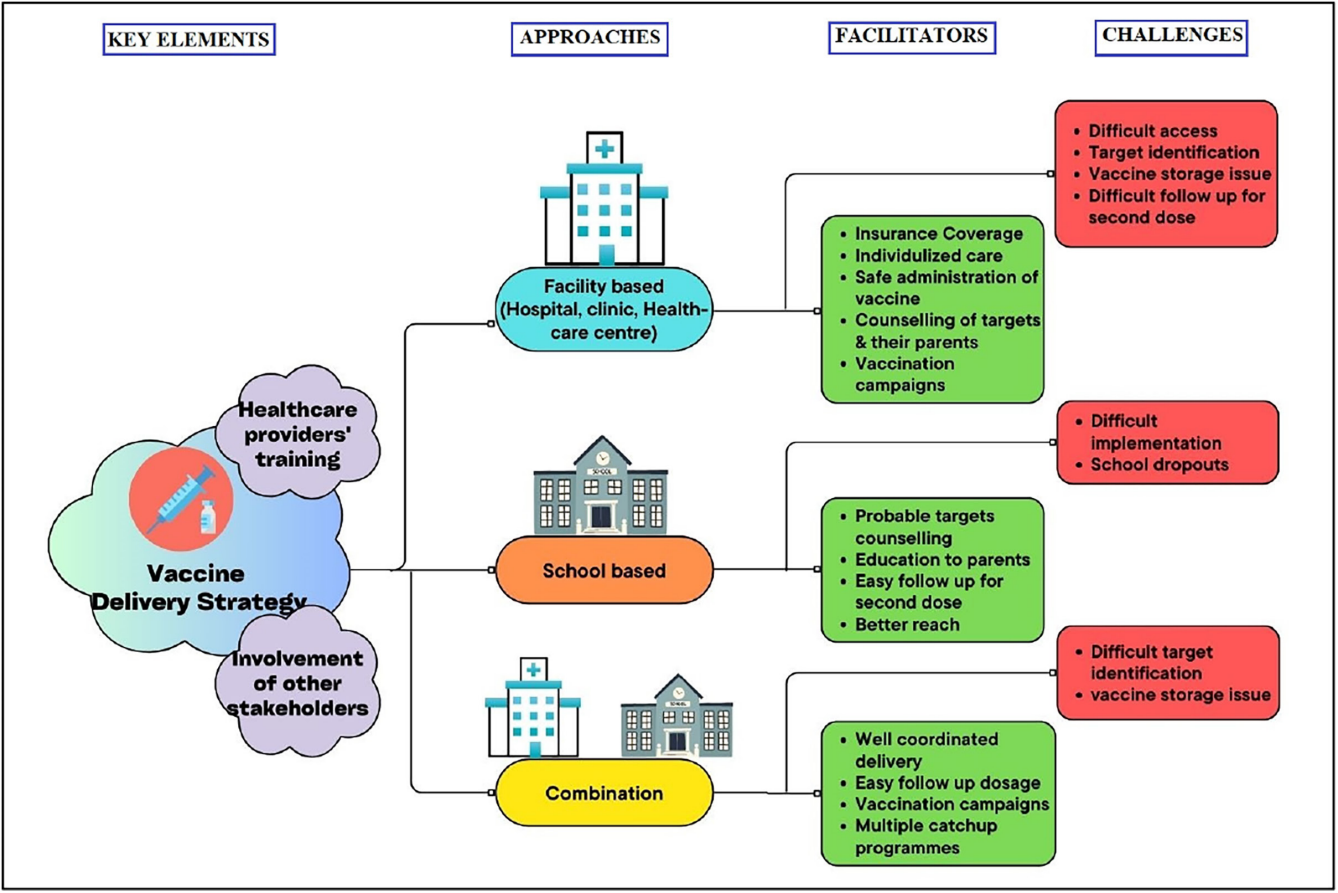
Facilitators and challenges of vaccination delivery strategies observed from various countries.

Few challenges have been identified during several vaccination drives in various countries across the globe (Figure 3).

These can be listed as:

- Health system‐related challenges
- Vaccine‐related challenge
- Individual‐related challenges

#### 3.6.1 Health system‐related challenges

Current experience illustrates that the introduction of HPV immunization requires extensive hard work and will, strong political backing, and adequate financial resources.

##### Delivery strategy

Countries need to make numerous critical decisions ensuring vaccine delivery, together with the primary and other vaccination sites, the timing and period of delivery and if the delivery requires to be integrated with other health or community services. Strategies should be modified over time and should be supplemented with facility and routine community outreach services as secondary sites for out of school (OOS) girls and for school girls who missed a dose at school.^10^ Careful attention to the timing of delivery is crucial to ensure that it does not gets overlapped with exams or hampered by rainy seasons or other barriers.^37^

##### Getting the message out

Targeted public health efforts must consider exploring internet together with social media as potential information distribution platforms, as evident from vaccination program of Switzerland. Factors related to vaccine hesitancy for instance lack of conviction in health authorities, vaccine hesitant doctors, perceived “newness” of vaccines, uncertainty about its benefits and myths that the vaccine may cause long‐term side effects also played role in low HPV vaccination rate in France.^21^ Though awareness for cervical carcinoma was better in Uzbekistan due to well‐coordinated program implementation, findings highlighted the requirement for clear, accessible, and credible information on the evidence base and safety of the HPV vaccine in order to be confident in support for an HPV vaccine as the knowledge and understanding gaps exist for health workers and the public related to HPV, cervical carcinoma and the HPV vaccine. As evident, inadequate sex education, especially in schools, contributed to insufficient knowledge about cervical carcinoma and vaccination among girls and their parents in Japan. In case of adverse events following immunization (AEFI), prompt and thorough investigation as occurred in Brazil is crucial for sustaining confidence and avoiding vaccine hesitance.^37^

##### Achieving high and equitable coverage and minimizing dropouts

Countries employing census records to estimate the target population are plagued by out‐of‐date census data frequently altered by internal migration and/or cross‐border flow of migrants or refugees or by girls shifting residence/school in single academic year. Reliance on school enrolment data can work well for grade‐based approaches but inefficient for age‐based programs and also promotes the risk of missing significant numbers of OOS girls, if school attendance is short.^10^ As evident globally, there is ~20% drop in number of girls receiving the second dose. Numerous factors have been recommended as the causes for the failure of girls to complete the immunization, including poor tracking systems, failure to provide apposite refresher training for the second dose while using campaign strategies, deficiency in proper tracking of girls' switching to another school and no particular reminder to get the final dose.^37^

##### Multisector coordination

Community leaders and political individuals play key role in identifying circulating misconceptions and controlling it via responding to them with correct messages. HPV vaccine coverage in France remained lower than in most other high‐income nations due to lack of political and strategic coordination by the Regional Health Agencies.^10^ In Japan, the government was slow to review the evidence while anti‐vaccine pressure became louder. Also, there was lack of information together with confidence regarding the effectiveness of HPV vaccine even among obstetricians and gynecologists. On contrary, in several countries (e.g., Senegal, Bolivia, and Zambia), the use of mobile apps for connecting health workers across the nation has facilitated the rapid identification and also in addressing of rumors and the plans were devised to respond to the most common issues by participating in TV and radio shows.^37^

#### 3.6.2 Vaccine‐related challenges

##### Vaccine type

Previously a quadrivalent HPV vaccine (Gardasil, HPV types 6, 11, 16, and 18) and a bivalent vaccine (Cervarix, HPV types 16 and 18) were licensed by most of the countries,^9^ which provided protection against HPV types which are accountable for most of the HPV carcinomas (~70%). Although presently a 9‐valent HPV vaccine having similar composition to the quadrivalent vaccine, using virus‐like particles (L1 protein of HPV 6, 11, 16, 18, 31, 33, 45, 52, and 58) have been licensed in many countries. With minimal additional cost and harm, the nonavalent HPV vaccine includes five additional high‐risk strains thus provides better protection (~90%) against HPV subtypes than the quadrivalent and bivalent counterparts do^38^ (Table 1). Thus, an efficient vaccination drive with the 9‐valent vaccine would possibly lead to further reduction of HPV‐associated carcinomas. The 9‐valent HPV vaccine could prove to be more cost‐effective than the existing quadrivalent HPV vaccine in female‐only vaccination programs based on the cost per dose.

##### AEFI

The most frequent adverse reactions observed postvaccination are local reactions such as injection‐site pain (mild or moderate), swelling with erythema and systemic adverse effects like fever in 4% of the recipients. No critical vaccine‐related adverse events have been reported yet.^39^ The most common adverse reaction was headache, fever, myalgia, nausea, dizziness, pain, swelling, erythema, pruritus, and bruising [Merck]. All reported AEFI cases in all the studies are appropriately managed and patients recovered within short duration. Although most of the reactions were of mild to moderate severity and were not long lasting [GSK], but may result into vaccine hesitancy among the participants if social media distributes these matters through wrong approaches.

##### Vaccine cost and supply

Middle‐income countries deprived of access to Gavi are predominantly challenged to keep decent monetary space in their budgets for HPV vaccine at present prices and in nations with especially huge populations. LMICs can also consider supply constraints to limit their capability either to introduce the vaccination or likelihood of series completion. The cost of delivery is the other challenge if there is no existing school health program into which HPV immunization can be simply integrated. Conducting outreach visits to schools once or twice a year, training teachers, informing parents and obtaining consent whenever required are extra costs in school‐based programs.^37^

#### 3.6.3 Individual‐related challenges

##### Age and dosage

For HPV, although new evidences suggest that even a single dose of vaccine may confer a durable immunologic response, WHO presently endorses a 2‐dose schedule with a 6‐month interval between doses is recommended for individuals receiving the first dose before 15 years of age. An off‐label single‐dose schedule can also provide a comparable efficacy and durability of protection to a 2‐dose regimen as recommended by WHO's independent expert advisory group, SAGE in April 2022.^40^ This could be revolutionary for the prevention of disease in low‐resource settings where the cervical carcinoma burden is relatively high and access to multiple doses of a vaccine is more difficult, as a 1‐dose schedule could reduce costs, simplify vaccine delivery and expand access to HPV vaccine compared to multidose schedules. However, immunocompromised individuals, including individuals with HIV, should receive 3 doses if feasible or at least 2 doses.

##### School drop outs

Although average coverage of HPV immunization can be accomplished using school‐based approaches, though, ensuring equitable access to HPV vaccine for OOS girls are needed that can be efficiently and effectively delivered alongside school‐based HPV vaccination drives which presents its own unique challenges. OOS girls, in general, belong to families with fewer resources and from more rural areas, which coincides with the risk factors associated with early marriage, age‐discordant relationships, and frequent sexually transmitted infections including HPV.^41^ For these concerns, the WHO has also highlighted the requirement for the development of complementary HPV vaccination strategies to identify, communicate with and access OOS girls. Communication strategies targeting OOS girls might utilize interactive communications, SMS reminders, radio announcements, community leaders, CHWs, school administrators or peers to identify and invite OOS girls to attend schools on the day of vaccination to get HPV vaccine alongside the enrolled girls. In national HPV vaccination programs in Rwanda, Colombia, Malaysia, and Mexico, approaches to reach OOS girls have utilized active tracing by CHWs and vaccination campaigns at local health centers,^41^ complemented with facility and routine community outreach services as secondary sites for OOS girls or school girls who missed a dose at school.

##### Information, education, and communication (IEC)/behavioral change communication (BCC)

Despite cervical carcinoma control guidelines are publicly existing, lack of communication strategy on cancer, awareness of risk factors, and coordinated prevention activities deficiency persist. Previous practice with IEC programs shows that only knowledge and awareness regarding HPV are not sufficient as the behavioral adoption is primarily influenced by the external environment, local context and community. Thus, targeted IEC/BCC should be conducted to create favorable attitude toward cervical cancer vaccination.^42^ The best public awareness strategy should be introduced by a community organization that makes it possible to routinely invite the target women in a timely manner, with a message reminder 1 week before the appointment, culturally appropriate education brochure to bring down barriers pertaining to their particular race or language. Parent teacher association for awareness of various programs for vaccination, individual and family counseling for instance facilitated by the ASHA and other IEC/BCC activities such as providing information and education to individuals to promote healthy behaviors should be adopted.

### 3.7 Post implementation research priorities

There are few questions which need to be addressed for successful future vaccination drives and by achieving these priorities, HPV vaccination can be made a reality for all adolescents which will aid to eliminate cervical carcinoma and other HPV‐associated diseases.

These future priorities include:

#### 3.7.1 Effectiveness of 1‐dose regimen of vaccine

The latest international scientific and clinical evidences confirmed that a single dose provides comparable protection against HPV infection in healthy young women. SAGE's review concluded that 1‐dose of HPV vaccine offers solid protection against cervical carcinoma which is comparable to 2‐dose schedules.^40^ In an Indian study, vaccine efficacy against persistent HPV 16/18 infections was (95% CI) 95.4% with single‐dose, 93.1% with 2 doses, and 93.3% in 3 doses recipients and the frequency of incident HPV 16 and 18 infections up to 7 years of follow‐up was similar in all the groups.^43^

Antibody responses after 1 dose in the Dose Reduction Immunobridging and Safety study (DoRIS) held in Tanzania and high efficacy of 1 dose up to 11 years against HPV16 and HPV18 was observed.^44^ In this line, Australia has also recommended to move from 2 doses to a single dose of the nonavalent HPV vaccine for routine immunization under the NIP from February 06, 2023. This is particularly important for LMICs and LICs where the resources are low and follow‐up vaccination is difficult.

#### 3.7.2 Vaccination among male population

HPV infection does not exclusively impact women, in fact it is also associated with anogenital carcinomas in men, specifically anal and penile and also vaccinating males is crucial to reduce the transmission of HPV to females. Pan‐gender vaccination programs are currently being adopted by many developed countries,^45^, ^46^ as multiple models have suggested that vaccinating both men and women is more advantageous than vaccinating only females.^47^, ^48^, ^49^, ^50^, ^51^ It is also evident that the awareness on HPV and vaccine is quite poor among men who have sex with men (MSM).

#### 3.7.3 Developing new HPV vaccines

Current HPV vaccines provide protection against the most common subtypes of HPV that cause cervical carcinomas. However, there are other types of HPV that can cause other HPV‐associated carcinomas, for example, anal and oropharyngeal cancer. Thus, developing new HPV vaccines that protect against a wider range of HPV types is an important priority for the future.

#### 3.7.4 Expanding access to vaccination

There are a number of barriers to HPV vaccination, such as cost, lack of awareness, and vaccine hesitancy. Addressing these barriers is essential to increasing vaccination coverage rates. HPV vaccination rates remain low in many countries, particularly among certain populations such as boys and young men, and adolescents from low‐income families. Efforts to increase vaccination rates should focus on reaching these populations and overcoming the barriers that prevent them from getting vaccinated. HPV vaccination should be made available to all adolescents, regardless of their ability to pay. This includes ensuring that vaccination is covered by insurance and that there are no financial barriers to getting vaccinated.

#### 3.7.5 Initiating vaccination at younger ages

The HPV vaccine is relatively more effective when it is given before a person is exposed to HPV. Therefore, it is important to initiate vaccination at younger ages, such as at age 9 or 10 to protect adolescents from HPV infection and the associated cancers.

#### 3.7.6 Providing catch‐up and follow‐up vaccination

Adolescents who miss the opportunity to be vaccinated during routine school‐based programs should be offered catch‐up vaccination. This is especially important for adolescents who are at high risk of HPV infection, such as those who are sexually active or who have a history of sexually transmitted infections. Also, campaigns should be arranged for follow‐up vaccination.

#### 3.7.7 Promoting awareness and education

Many people are not aware of the benefits of vaccination or the importance of getting vaccinated. Therefore, it is important to promote awareness and education about HPV vaccination so that people can make informed decisions about their health.

#### 3.7.8 Evaluating the long‐term effectiveness of HPV vaccination

HPV vaccination has been observed to be efficient in preventing HPV infection and the associated cancers. However, further research is required to evaluate the long‐term effectiveness of vaccination. In Spain, the first of those vaccinated (quadrivalent vaccine) cohorts, turned 25 years in 2018 when the screening was performed. There was a substantial decrease in the prevalence of HPV‐6 (0% vs. 1.3%; vaccinated vs. non‐vaccinated group respectively), HPV‐16 (2.4% vs. 6.1%), and HPV16 linked cytological abnormalities; low‐grade squamous intraepithelial lesions (2.94% vs. 18.7%) and high‐grade squamous intraepithelial lesion (0% vs. 40%).^52^ Among Swedish girls and women (10–30 years) also, quadrivalent HPV vaccination was associated with significantly reduced risk of invasive cervical carcinoma at the population level.^53^ In Finland as well, 10 years postvaccination with bivalent vaccine, continued efficacy against CIN3+ was reported.^54^ Cervical cancer rates decreased at population level in the United States also postvaccination, especially in younger women.^55^ Similarly, a substantial reduction in cervical cancer and incidence of CIN3 in young women has been observed after the introduction of the HPV immunization program in England, especially in females who were vaccinated at 12–13 years of age.^56^

#### 3.7.9 Screening policies post HPV vaccination program

The bivalent and quadrivalent HPV vaccines do not provide protection against all high‐risk genotypes of HPV, so cervical cancer screening is still important after introducing vaccination for the time being. National screening program exists in many countries who have introduced HPV vaccine into their NIP. It is recommended that screening (Pap smear test) should be initiated from 30 to 65 years of age in normal women, with regular validated HPV test every 5–10 years and from 25 years for women with HIV with HPV screening at every 3–5 years. A test to triage the HPV positive women (e.g., VIA) is essential before treating HIV‐positive women.^57^

An optimal cervical carcinoma screening regime for females vaccinated with all 3 doses was proposed in 2017 from a US model based‐analysis of benefits and costs, recommended that screening schedule could be revised to start later with decreased frequency, with either cervical cytology or HPV testing alone at every 5 years for individuals vaccinated with bivalent or quadrivalent vaccine and only primary HPV testing at every 10 years beginning from age 30 to 35 for women vaccinated with nonavalent vaccine.^58^

#### 3.7.10 Development of newer diagnostics

While numerous new screening tools have been proposed for HPV detection, an effective approach for viral load testing detects mRNA of E6 and E7 transforming genes.^59^ The E6/E7 mRNA expression might reflect the phase of HPV infection, and its positive pattern might also forecast the progression of CINs, thus efficiently improving the diagnostic sensitivity and specificity of cervical carcinoma and precancerous lesions, thereby avoiding over‐examination. Devices for self‐collection of urine which are licensed for gender neutral usage, have a high acceptance globally. One of the reasons for younger women's low visiting frequency to gynecology clinic is because of the uncomfortable sampling procedure for HPV testing. Thus, it is essential to use a noninvasive method, for example, urine testing, to early screen HPV infection rather than the conventional invasive methods, which could allow frequent samplings along with the sampling of large populations participating in HPV vaccination drives. The approach can be further explored as one of the most effective noninvasive methods for early detection of HPV through cytological diagnosis or sequencing in young women.^60^, ^61^

#### 3.7.11 Role of herd immunity

Studies suggest that HPV vaccination provide herd protection; that is, indirect protection to those who remain susceptible and have not been vaccinated, owing to a reduced prevalence of infections.^62^ HPV vaccination produce important herd effects for the anogenital warts associated types (HPV 6 and 11) as well. Thus, better understanding of herd effects leading to substantial long‐term reductions in HPV infection, is crucial to help inform future vaccine policy decisions such as the inclusion of boys in vaccination programs.^63^

**Table:** 

**Summary of facilitators, barriers, and future priorities for implementation of HPV vaccination drives** **Facilitators** Health care providers' training is essential. Involvement of other stakeholders is of utmost priority. Counseling of targets and their parents must be carried out Individualized care is important. Vaccination campaigns should be arranged. Multiple catchup programs are highly recommended. **Barriers** Targets' identification for vaccination is hard. Follow‐up for second dose is difficult. Vaccine storage issue may occur due to low temperature requirements. Record of school dropouts needs to be maintained. **Future priorities** Multisector coordination must be ensured. Information, education, and communication and behavioral change communication. Campaigns for follow‐up dosages must be arranged. Effectiveness of 1‐dose regimen of vaccine needs to be evaluated. Development of new multivalent HPV vaccines. Evaluation of long‐term effectiveness of HPV vaccination. Vaccination of male population.

## 4 LIMITATIONS OF THIS REVIEW STUDY

However, this review has utilized a step‐wise strategy and the included studies were subjected to in‐depth analysis applying challenges in HPV vaccination strategies, the study has its limitations and its findings should be considered in the light of these, such as the search was limited to the items published in English. Although to mitigate this issue, we have uniformly employed our eligibility criteria and used a comprehensive list of keywords and various databases but it is likely that some relevant studies might have been missed.

## 5 CONCLUSIONS AND FUTURE DIRECTIONS

With existing prevention and treatment modalities, cervical carcinoma prevention and eradication is now possible. Though there are several methods to prevent cervical carcinoma, prevention by vaccination is emerging as the most effective option. However, it is clear that cervical cancer vaccine is not an immediate panacea and cannot substitute the cervical carcinoma screening and a combined policy of high‐coverage HPV vaccination of girls (9–14 years) and affordable screening practices for women (35–45 years) can potentially reduce its load as a public health problem. With effective implementation of best vaccination practices, strategies can be scaled up more broadly and introduction of an effective approach for high vaccine coverage may offer the easiest and effective way to control cancer incidence in the upcoming future. The present review is relevant for strengthening vaccination programs to achieve improved immunization coverage. Easy, equal and free access to vaccination; well‐coordinated delivery services, and increased knowledge are prerequisites in the program's success. Thus, by identifying challenges and learning from other countries nation vide implementation could be achieved successfully.

## AUTHOR CONTRIBUTIONS

**Sumit Aggarwal:** Conceptualization (lead); supervision (lead); writing – review and editing (equal). **Pragati Agarwal:** Conceptualization (supporting); visualization (lead); writing – original draft (lead); writing – review and editing (supporting). **Nivedita Gupta:** Conceptualization (supporting); writing – review and editing (lead).

## FUNDING INFORMATION

This research did not receive any specific grant from funding agencies in the public, commercial, or not‐for‐profit sectors.

## CONFLICT OF INTEREST STATEMENT

The authors have no interest to declare.

## Data Availability

Data sharing not applicable to this article as no datasets were generated or analyzed during the current study.
